# Numerical and Experimental Study of Cross-Sectional Effects on the Mixing Performance of the Spiral Microfluidics

**DOI:** 10.3390/mi12121470

**Published:** 2021-11-29

**Authors:** Omid Rouhi, Sajad Razavi Bazaz, Hamid Niazmand, Fateme Mirakhorli, Sima Mas-hafi, Hoseyn A. Amiri, Morteza Miansari, Majid Ebrahimi Warkiani

**Affiliations:** 1 School of Biomedical Engineering, University of Technology Sydney, Sydney, NSW 2007, Australia; omid.rouhi1989@gmail.com (O.R.); sajad.razavibazaz@student.uts.edu.au (S.R.B.); fateme.mirakhorli@student.uts.edu.au (F.M.); 2 Department of Mechanical Engineering, Ferdowsi University of Mashhad, Mashhad 91779-48974, Iran; niazmand@um.ac.ir; 3 Micro+Nanosystems & Applied Biophysics Laboratory, Department of Mechanical Engineering, Babol Noshirvani University of Technology, P.O. Box 484, Babol 47148-71167, Iran; sima.mashafi@gmail.com (S.M.-h.); aamirihoseyn@gmail.com (H.A.A.); mmiansari@nit.ac.ir (M.M.); 4 Department of Cancer Medicine, Cell Science Research Center, Royan Institute for Stem Cell Biology and Technology, Isar 11, Babol 47138-18983, Iran; 5 Institute of Molecular Medicine, Sechenov University, 119991 Moscow, Russia

**Keywords:** 3D printing, spiral micromixers, dean flow, trapezoidal cross-section, mixing index, convection and diffusion

## Abstract

Mixing at the microscale is of great importance for various applications ranging from biological and chemical synthesis to drug delivery. Among the numerous types of micromixers that have been developed, planar passive spiral micromixers have gained considerable interest due to their ease of fabrication and integration into complex miniaturized systems. However, less attention has been paid to non-planar spiral micromixers with various cross-sections and the effects of these cross-sections on the total performance of the micromixer. Here, mixing performance in a spiral micromixer with different channel cross-sections is evaluated experimentally and numerically in the *Re* range of 0.001 to 50. The accuracy of the 3D-finite element model was first verified at different flow rates by tracking the mixing index across the loops, which were directly proportional to the spiral radius and were hence also proportional to the Dean flow. It is shown that higher flow rates induce stronger vortices compared to lower flow rates; thus, fewer loops are required for efficient mixing. The numerical study revealed that a large-angle outward trapezoidal cross-section provides the highest mixing performance, reaching efficiencies of up to 95%. Moreover, the velocity/vorticity along the channel length was analyzed and discussed to evaluate channel mixing performance. A relatively low pressure drop (<130 kPa) makes these passive spiral micromixers ideal candidates for various lab-on-chip applications.

## 1. Introduction

With the progress in device miniaturization technology, micro-platforms have drawn significant attention in various realms, including environmental science, chemistry, and therapeutics [1]. Among them, microfluidics, which manipulate fluid flow inside a microchannel, has progressed considerably due to its privileges such as enhanced control over the experiment, reduced reagent consumption, and the ability to fabricate compact and portable devices [2]. Accompanied by immense improvements in such platforms, their application has flourished drastically in several fields, including in drug delivery [3], cell isolation and lysis [4,5], polymerization [6], DNA amplification [7], and crystallization [8]. In many microfluidic devices, the sample should be mixed with a reagent prior to experimentation. However, the flow regime in most of these microfluidic devices is laminar, where slow mixing occurs by pure diffusion, making efficient mixing challenging. To address this concern, diverse micromixer designs have been proposed and optimized. Thus, once the purpose of an experiment has been identified, the optimum design and fabrication method should be selected based on the operational requirements and the available equipment of the practiced application.

Generally, micromixers are divided into two categories based on the external energy usage aspect: active and passive [9]. Active micromixers employ various acoustic, electric, magnetic, or optic forces to enhance the mixing efficiency in a short period of time. However, they have some serious drawbacks, such as detrimental effects on the biological reagents or troublesome integration with other microfluidic devices since they are inherently slow, and matching their flow rates with other microfluidic components is challenging [10]. On the other hand, passive micromixers rely on optimizing the channel geometry, creating chaotic advection, or enhancing the molecular diffusion to boost the mixing efficiency. In this regard, their simplicity, low-cost fabrication, and ease of integration with other lab-on-a-chip platforms provide better functionality for practical applications. Hence, passive micromixers have become of interest and are considered to be a viable option for commercialization [11].

In passive micromixers, diffusion and advection are the two main mechanisms that can be used to control mixing inside a micromixer. Molecular diffusion is inherently very slow. There are limited methods that can be used to increase the efficiency of this method: lamination or split and recombine (SAR), although they are not as productive as advection-based mixing mechanisms. On the other hand, advection has more impact. To enhance the mixing, different types of micromixers use chaotic advection concepts, such as staggered herringbones [12], serpentines [13,14], and spirals [15]. The idea of combining different mixing units has also been tested [16]. Due to the centrifugal force inside of a curved channel, the maximum velocity point in the parabolic profile of laminar flow is drawn towards the outer wall, creating a velocity gradient and consequently an adverse pressure gradient along the channel cross-section. These changes cause two counter-rotating vortices that are perpendicular to the mainstream that are called Dean vortices. The Dean number indicates how strong those vortical patterns are and is proportional to the Reynolds number (Re), hydraulic diameter ($D_{h}$), and radius of curvature (R) [17,18].

In addition to the significant impact of high mixing efficiency in a sample preparation step during an experiment, incubation time is of great importance in numerous applications, including during protein coating and crystallization [19,20,21]. This can be explained by the fact that for a complete reaction, two or more samples and reagents need to contact each other for a specific amount of time, and the only way to attain this is to increase the mixing length. Nevertheless, there are limitations in increasing the mixing length, such as extending the fluid path, increasing the pressure drop, and enlarging the channel footprint, which conflicts with miniaturization and portability. Therefore, there is a need to have a maximum mixing length in a minimum footprint, i.e., a compact, efficient micromixer [22]. In this regard, the spiral microchannels are proper candidates. This design not only satisfies the requirements of a compact micromixer but also has a relatively high mixing index and straightforward fabrication process.

Howell et al. [23] were the first to propose spiral-shaped mixer devices. Although the channel dimensions were big, they studied the vortex formation at different Reynolds numbers, channel aspect ratios, and initial radii. Expectedly, later studies have developed this structure for micromixers and have applied different techniques with various modifications to enhance the mixing efficiency. Moreover, a combination of varying mixing effects such as SAR and expansion with Dean vortices have been studied [24]. These effects were further characterized using confocal microscopy techniques, which presented a cross-sectional view of transverse Dean flow inside of the curved channel. Various geometrical features can influence the mixing quality. The angle between two inlets, for example, is an essential parameter in mixing processes. Zhang et al. [25] investigated this effect in the mixing performance of spiral micromixers both numerically and experimentally. The mixing results showed that the mixing index (MI) improved by increasing the inlet mixing angle, with the best angles being obtained between 90–180 degrees. As microchannel cross-sections are of great diversity, numerical methods are applied as an efficient comparative tool for various operational conditions. Kumar et al. [26] computationally investigated the fluid dynamics of a cross-section for a curved tube in contrast with a straight tube. Based on Reynolds number and Schmidt number (*Sc*), the study showed that mixing is enhanced at a moderate *Re* (~10) for a higher *Sc*, while this behavior does not apply to a *Re* in the order of 0.1. Another research study on the effect of the cross-section aspect ratio in spiral micromixers showed that mixing performance in high-aspect ratio micromixers is better for a wide range of *Re* ranging from 1 to 468 [27]. Furthermore, a parametric study was carried out on the mixing process in spiral micromixers [28]. This study discussed various key variables on mixing efficiency, such as initial radius and cross-section shape, where they compared the impact of inlet velocity between straight and spiral micromixers. They reported that the mixing performance increases when the initial radius decreases while the straight micromixer acts oppositely. Very recently, Wang and colleagues studied the mixing enhancement of various spiral structures, including Archimedean, Logarithmic, Hyperbolic, Golden, and Fibonacci spirals, and concluded that the Archimedean spiral has the best performance among the other types [29]. However, so far, the effects of channel cross-section on the total performance of spiral micromixers have not been investigated.

Alongside all of the parameters and functionalities of micromixers, finding the proper fabrication method is vital for device miniaturization [30]. The softlithography method has been widely used due to the channel transparency for live monitoring, biocompatibility, and gas permeability [31,32]. However, in this method, master mould fabrication requires several laborious processes such as photolithography or micro-milling, which are time-consuming and are mainly applied to the manufacture of planar devices. To address these issues, additive manufacturing or 3D printing has evolved as a promising method for fabricating complex devices. In this method, parts with intricate geometries can be manufactured with high precision [33,34].

The present work concentrates on analyzing the mixing qualities of several spiral micromixers with unconventional cross-sections. Using numerical and experimental results, we nominated the most efficient cross-section for flow field and performance analysis in terms of the mixing index and the pressure drop at the outlet. The experimentally validated numerical model was conducted using the 3D finite element method to solve the convection–diffusion equation as well as the Navier–Stokes momentum equation. In addition, the mixing qualities and pressure drops were evaluated across a wide range of Reynolds numbers. Then, the effect of the velocity field and Dean flow on the mixing mechanism was investigated in detail for several flow conditions. Finally, the vortical impact was explored as the fluid poured into each loop, leading to compact, efficient micromixer structures being obtained according to the required application and flow rates. The selected designs were compared with each other using the mentioned performance parameters. Consequently, a general map of the mixing index was obtained for the most appropriate design by changing the *Re* along the loops.

## 2. Materials and Methods

In the following, the proposed spiral micromixer structures, design, and fabrication process are described. After selecting the micromixer designs, their efficiency is evaluated at the same *Re* by employing numerical simulation. The underlying theories and governing equations for the current physics are discussed, and analysis tools are used to achieve the optimal design from a range of geometrical/operational features.

### 2.1. Spiral Designs and Fabrication Methods

**Figure 1:**
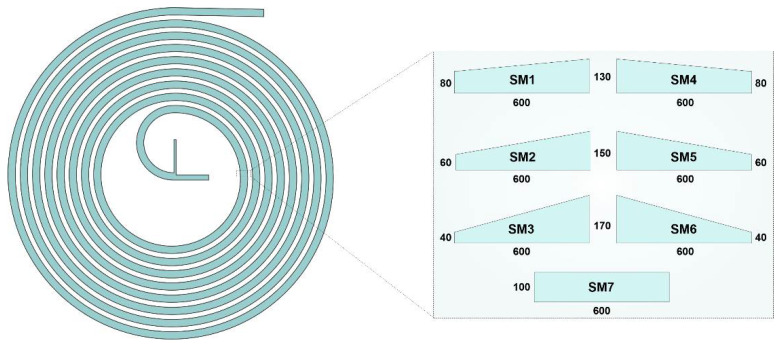
Schematic of the microchannel and the proposed cross-sections along with the applied boundary conditions. SM1 to SM3 in the left column are trapezoidal cross-sections before flipping when the outer wall is shorter, while SM4 to SM6 on the right column refer to mirrored ones with shorter inner walls. The cross-section hydraulic diameter and total fluid volume capacity of the micromixer are kept equal in all cases. All dimensions are in micrometers.

A spiral microchannel with trapezoidal cross-section was designed by our group [35] for CTC isolation and had a width of 600 µm, a 80 µm height in the inner wall, and a 130 µm height for the outer wall, which was previously modified here in terms of the height ratio, cross-section, and the position of the walls in order to investigate the effect of each factor on the mixing index. Here, two major changes have been made to the original cross-sectional design: First, the channel height ratio, being the larger height relative to the smaller one, has changed compared to the original design. Thus, the 80 μm wall was reduced to 60 μm and 40 μm, while the larger other wall helped to maintain a constant hydraulic diameter and volume (enabling valid comparison based on *Re*). Since the inner wall (80 µm) was modified to be shorter than the outer wall in the original pattern, the second step was to flip the channel cross-section, i.e., switching the position of the sidewalls. In the end, a rectangular cross-section was drawn in order to gain insight into the effect of the cross-section shape. Therefore, in all of the geometries, the key variable is channel cross-section. While the fluid flows through a curve, the inner/outer near-wall velocity decreases/increases due to the centrifugal force and the pressure gradient. The pressure variation enforces a secondary flow that is perpendicular to the main flow, which benefits the mixing process in high inertial fluid states. Hence, as illustrated in Figure 1, a total of seven cross-sections comprising outward and inward (mirrored) trapezoidal and rectangular geometries were studied to determine the most efficient structure.

All of the devices were fabricated using a high-resolution DLP 3D printer (Miicraft, Hsinchu, Taiwan) with a 32 × 57 × 120 mm^3^ printing volume and 30 µm XY resolution. The geometry was printed on one side and was then attached to a substrate (PMMA sheet) using a double-sided adhesive. The desired micromixer device was initially drafted using a commercially available CAD drawing software (SolidWorks 2019, Waltham, MA, USA) and was then exported in STL format. This STL format is suitable for 3D printer language. The Miicraft software (Version 6.1.0.t4, Miicraft, Hsinchu, Taiwan) was used to slice the file in the Z direction. The slicing in the Z direction (slice thickness) can be adjusted from 5 to 200 µm with an increment of 5 µm [36,37].

The complexity of the channel assists the user in choosing the proper slicing option. A ramp or steps in geometries leads to a smaller slice thickness, while orthogonal or planar channel structures result in a higher slice thickness. Afterward, the file, which was previously sliced, was sent to the 3D printer, which has a UV wavelength of 358–405 nm UV. The UV light was projected from the bottom of the resin bath, which had already been filled with BV-007 resin. The UV light passed from a transparent Teflon film and cured the resin on the picker of the 3D printer. As soon as one layer formed on the picker, the stepper motor relocated one layer and started printing another layer. This process continued until the whole part was printed successfully. Once the part was printed, it was removed from the picker and was washed carefully with isopropanol three times. As a post-curing step, the channel was placed in a UV chamber with a wavelength of 405 ± 5 nm. Finally, the tubes were mounted on the parts using tweezers. The inlet and outlet holes were printed in a way where they could keep the tubes firmly together to eliminate any chance of leakage [38]. The parts printed via this method were of high quality; we previously characterized the surface characteristics of the fabricated parts and measured and reported the surface roughness (*Sa*) of the parts that were printed via this method. Its value was less than 300 nm, indicating that surface roughness does not disturb channel performance and that it is appropriate for various microfluidic applications [39].

### 2.2. Numerical Model

Newtonian fluid flow is considered to be steady, incompressible, and laminar; hence, general governing equations for fluid mixing include the continuity of mass and momentum [40], which are shown as:

(1) ∇·V=0

(2) $$\frac{\partial V}{\partial t}+\rho \left(V\cdot \nabla \right)V=-\nabla P+\mu \nabla^{2}V$$

where V, P, ρ, and μ are the fluid velocity field, pressure, density (998 kg/m^3^), and dynamic viscosity (8.9 × 10^−4^ Pa∙s), respectively. The boundary conditions include constant velocity inlets ($V_{in,1}=U_{1}$ and $V_{in,2}=U_{2}$), the pressure outlet ($P_{out}=0$), and the no-slip walls for all cases. Since the hydraulic diameter and therefore the flow volume does not change in the different investigated structures, the inlet velocities differ throughout the *Re* range. The convection–diffusion equation governs the species concentrations (c) for fluids with a diffusion coefficient of D (2 × 10^−9^ m^2^/s), which is similar to other works [26,27,28,35,40,41,42] and can be determined as:

(3) $$\frac{\partial c}{\partial t}+\left(V\cdot \nabla \right)c=\frac{1}{ReSc}\nabla^{2}c$$

where Re is the Reynolds number of the channel and where Sc is the Schmidt number, which defines as the ratio of momentum to mass diffusivity. The concentrations are considered as 0 and $c_{0}=1$ mol/m^3^ for the inlet boundary condition, where the average molar fraction of $\bar{c}=0.5$ mol/m^3^ is referred to as complete mixing. Consequently, the coupled governing equations were solved in COMSOL Multiphysics 5.3a (COMSOL, Burlington, MA, USA) by applying the proper boundary conditions. Mixing quality was evaluated by the mixing index criterion that was defined based on the deviation from $\bar{c}$ [43]:

(4) $$MI=1-\sqrt{\frac{1}{n}\overset{n}{\underset{i=1}{\sum }}{\left(\frac{c_{i}-\bar{c}}{\bar{c}}\right)}^{2}}$$

where MI, n, $c_{i}$, and $\bar{c}$ are the mixing index, number of sample points, concentration of the species, and average concentration, respectively.

### 2.3. Sample Preparation

In order to visualize the mixing process through bright field imaging, food coloring (Queen Fine Foods, Alderley, Queensland, Australia) was used. To prepare the sample, 1 mL from each dye was dissolved in 49 mL of MACS buffer (Miltenyi Biotec, Bergisch Gladbach, Germany) containing phosphate-buffered saline (PBS) and 2 mM ethylenediaminetetraacetic acid (EDTA) supplemented with 0.5% BSA, and 0.09% sodium azide.

### 2.4. Experimental Results

An inverted microscope (IX73, Olympus, Tokyo, Japan) equipped with a CCD camera (DP80, Olympus, Tokyo, Japan) was used for brightfield imaging. Samples (green and red) were filled into the 50 mL plastic Syringe (BD, Franklin Lakes, NJ, USA) and were injected into the micromixer using a syringe pump (Chemyx Fusion 200, Stafford, TX, USA).

## 3. Results and Discussions

### 3.1. Grid Study

**Figure 2:**
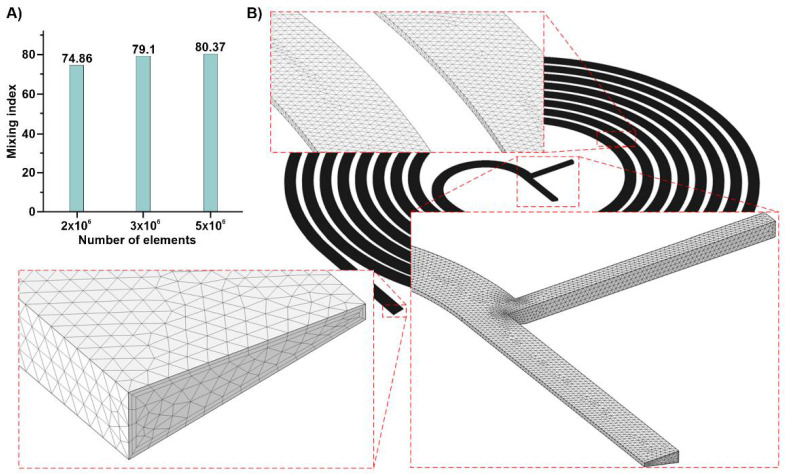
The information for the applied grids. (**A**) The grid independence study; (**B**) The selected grid is shown in detail in several regions of the channel.

Grid independence is of significant importance and directly affects the accuracy of the results. The quality of the results obtained with a small number of elements is not high since only a small number of discrete points within the domain are evaluated and solved. On the other hand, simulation with an increased number of elements results in more accurate data, increasing computational time. Consequently, a trade-off between the number of elements and calculation time is required. Accordingly, an initial simulation was performed with three grid numbers to evaluate the effect of the element size on the mixing index in a trapezoidal spiral micromixer. Figure 2A shows a big gap between the results for the 2 × 10^6^ and 3 × 10^6^ mesh numbers. Subsequently, another simulation was performed with 5 × 10^6^ elements, and the difference between the two results is negligible. Henceforth, the latter was selected for the simulations, and its details, such as tetrahedral elements and boundary layer cells, are demonstrated in Figure 2B.

### 3.2. Spiral Micromixers with Various Cross-Sections

Spiral microfluidic devices have shown great promise in the area of microfluidic because of their flexibility when parameters such as cross-section, initial radius, and the number of loops change. Additionally, changing the above parameters can flexibly affect enormous interactive forces and regimes inside a spiral microfluidic device. This feasibility leads to various applications for these types of devices such as mixing, focusing, and separation, all of which being applications where the channel cross-section plays the most critical role in their functionality [44,45]. In this regard, Figure 1 shows the changes that were applied in order to determine the effect of the cross-sectional parameters in a spiral micromixer based on the initial geometry.

**Figure 3:**
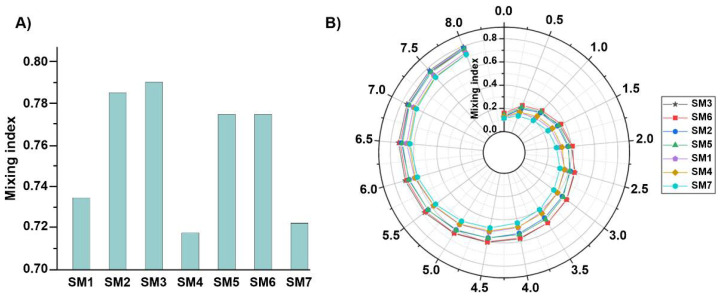
Evaluation of the mixing performance at *Re* = 5 for the proposed micromixers based on (**A**) final and (**B**) along-the-loop mixing index. The impact of height ratios on mixing quality is more significant than the outward/inward position of the shorter wall.

The numerical model was implemented on a variety of spiral micromixers that had been discussed earlier to capture the slightest concentration gradient and to identify the most efficient structure. The final mixing index at Re=5, which is shown in Figure 3A, is generally known as having the least efficient Reynolds number, highlights that SM3 offers the best overall mixing qualities. Therefore, the higher the height ratio is, the better the mixing index becomes, and this is due to the broadened centrifugal force and the secondary flows. As a result, when the fluid moves from the shorter wall to the larger wall, the higher wall height ratio creates a greater surface area near the longer wall, allowing sufficient space for secondary flows, increasing the chance of mixing. Upon that, the cross-section benefits from the forces acting on in- or outward direction of the fluid. Moreover, due to the significantly smaller height ratios, the mixers with an 80 µm height (SM1 and SM4) perform somewhat similarly to the rectangular micromixer (SM7), exhibiting the least predictable mixing performances.

In Figure 3B, the mixing index growth along the spirals suggests that the performance of these mixers rises gradually as the fluids flow through each loop. The difference between the designs with the high height ratio (SM3 and SM6) and other geometries is evident from the initial loops, which is remarkable until the sixth loop. In contrast, poor performance was observed for cases with a low height ratio, which is evident from the beginning of the spiral. Nevertheless, another trend that was observed shows that the outward channels outperform the inward channels. Overall, the height ratio is far more influential than the inward/outward positioning of the walls. Final evaluations confirm SM3 as the most efficient structures. Thus, it was nominated as the micromixer structure of choice.

### 3.3. Numerical Validation

**Figure 4:**
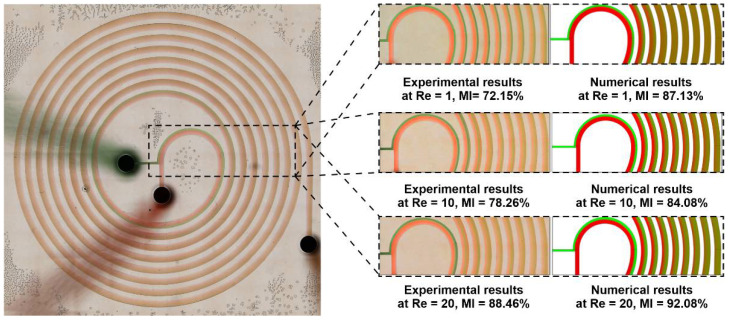
Numerical model validation and assessment with experiments at *Re* = 1, 10, and 20 using SM3. An overall view of mixing performance obtained via experimental observation (**left**) and numerical simulation (**right**). Fluids in the channel are shown in two distinct colors: green and red. The numerical errors seem to be insignificant, and the model agrees well with experiments in different situations.

Figure 4 depicts the comparison of the mixing trends between the numerical method and experimental runs of the nominate channel (SM3). In this experiment, the samples were loaded into BD plastic syringes with Luer-Lok tips, were mounted on a syringe pump (Chemyx Fusion 200, Stafford, TX, USA), and were then loaded into the channels via Tygon tubing. We used tubing with an inner diameter of 0.02 and an outer diameter of 0.06 inches. For the visualization of the mixing process in the bright field, food coloring (Queen Fine Foods, Alderley, Queensland, Australia) were used. The inlet flow rates were set to 12.5 and 9.5 µL/min for *Re* = 1, 125 and 95 µL/min for *Re* = 10, and 250 and 190 µL/min for *Re* = 20 for each inlet. The results for the three nominal Reynolds numbers show similar mixing behavior before, at, and after the critical *Re* = 5. The snapshots were captured after a sufficient amount of time, meaning that the flow represents a steady state. This agreement between both methodologies is satisfactory in the initial loops wherein the widthwise concentration gradient is relatively more intense. Moreover, the mixing that takes place in the earlier stages occurs at the two-fluid interface, producing a narrow area. This mixing interface is shifted towards the center of the spiral with the *Re* growth due to the involvement of the Dean vortices. Accordingly, an efficiently mixed flow is obtained with fewer loops and when using a greater velocity. To quantitatively validate numerical simulations, Fiji, an image processing software, was used to extract the values of the mixing index from the experimental snapshots. To this end, a line that was parallel to the channel width at the inlet and outlet of the microchannel was drawn, and the grayscale values of the image were extracted. These values have been normalized using Equation (5).

(5) $$I_{i}=\frac{I_{i}^{*}-I_{min}^{*}}{I_{max}^{*}-I_{min}^{*}}$$

Here, $I_{i}^{*}$ is the actual intensity, and $I_{min}^{*}$ and $I_{max}^{*}$ are the minimum and maximum intensity values at the channel inlet (unmixed fluid), respectively. Using these values, the experimental mixing index can be calculated using Equation (6).

(6) $$MI_{experimental}=1-\frac{\sqrt{\frac{1}{N}\sum_{i=1}^{N}{\left(\frac{I_{i}-\bar{I}}{\bar{I}}\right)}^{2}}}{\sqrt{\frac{1}{N}\sum_{i=1}^{N}{\left(\frac{I_{min,max}-\bar{I}}{\bar{I}}\right)}^{2}}}$$

where the pixel numbers in the inlet or outlet image along a line parallel to the channel width are represented by N, $I_{min,max}$ is either 0 or 1, and $\bar{I}$ is 0.5. Using the above formulas, the$\;MI_{experimental}$ for *Re* = 1, 10, and 20 was extracted and calculated and is shown in Figure 4. The difference between the values is mainly related to the fact that the mixing index in numerical simulations is evaluated along the channel cross-section, while in experimental snapshots, it is evaluated from the top view. In all, both the qualitative and quantitative comparison of the mixing process reveals that the numerical simulations agree well with experimental ones, proving that computational fluid dynamics is a proper approach for the investigation of the mixing process that occurs within micromixers.

### 3.4. Nominated Designs: A Comparison

**Figure 5:**
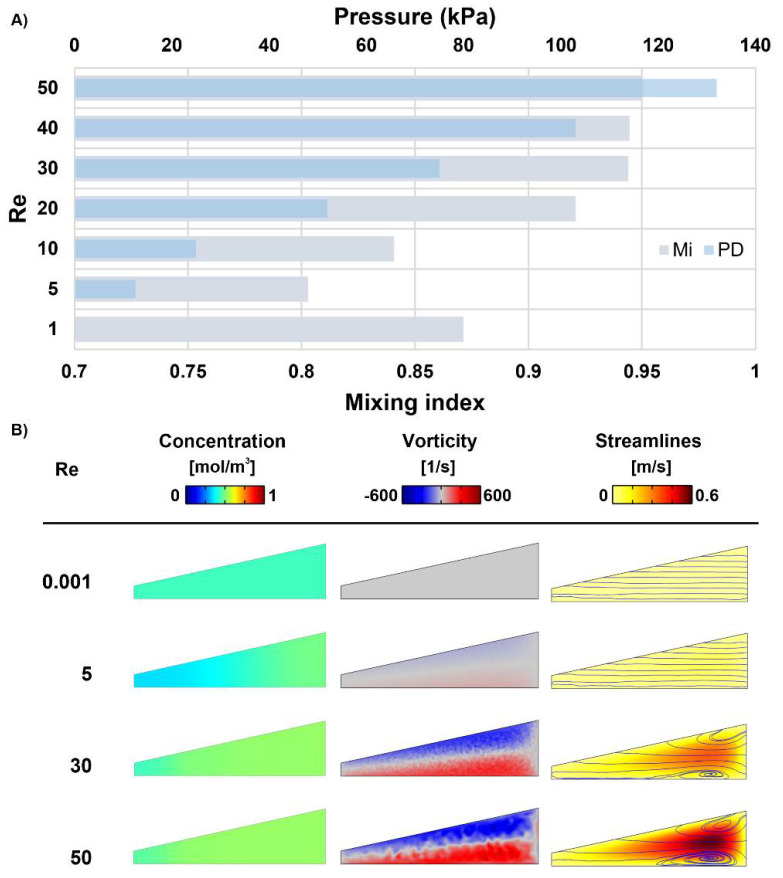
Mixing parameters evaluated at the final loop of SM3 in *Re* = 0.001–50. (**A**) Mixing index variations and pressure drop changes achieved by altering flow rates; (**B**) Concentration distribution, vorticity intensity, and fluid streamlines at the final loop of the nominated design for the selected flow conditions.

As observed in Figure 5, the nominated micromixer was analyzed in a wide range of *Re* = 0.001–50 to highlight the performance advantages in different working conditions. More cases were analyzed between *Re* = 1–10 because there were more fluctuations and sensitivity during this period. There is a negative balance spot between the diffusion and convection regimes at *Re* = 5. Thus, Figure 5A confirms *Re* = 5 as the critical condition with the least efficient mixing process, which still demonstrates a suitable amount of mixing at 80%. Before this *Re* and hence at lower flow velocities, there is more time for the fluid molecules to disperse and diffuse onto the interface. In general, the available time reduces by increasing the *Re*, and the vortices/secondary flows are not initially strong enough to compensate for the time reduction. As a result, the mixing index is poor until the cross-sectional flow can overcome this weakness after *Re* = 5, leading to a rapid increase in the mixing quality. In conclusion, starting from a *Re* = 0.001 with M.I. = 88%, the mixing quality first decreases, and the critical *Re* increases afterwards. Nevertheless, in the present case, the mixing efficiency usually saturates after a flow rate is assigned to *Re* = 30. Furthermore, the flows with the highest flow rate create the best mixing, with mixing efficiencies of up to 95% being demonstrated at *Re* = 50. However, this mixing quality also causes the highest pressure drop, which is depicted in Figure 5A. Although the micromixer performs appropriately in the studied velocity range, the optimum efficiency is achieved at moderate Reynolds numbers (*Re* = 20–40) without reaching the 100 kPa pressure-drop limit.

The influence of the *Re* is further confirmed by observing the concentration distribution, vorticity contours, and streamlines at the outlet in Figure 5B. The outlets have a uniform concentration distribution at *Re* < 5, where mixing is mainly guided by molecular diffusion; hence, the order of magnitude is small for the vorticity, leading to insignificant changes in the streamlines of the laminar flow. Therefore, both fluids are evenly mixed throughout the channels due to the low speed and sufficient time for molecular mixing. This uniformity, however, does not appear at a moderate *Re* of more than five since the molecular diffusion becomes less dominant and because the transition to the leading vortex effect occurs. By moving further away from that region, the mixing index starts to be much higher, and the concentration is not heterogenous all over the cross-section. Finally, the outer wall regions achieved better mixing due to the strengthened vortices at *Re* > 30 (*Re* = 30 is when the vortical pattern emerges) given the adequate total length/mixing time. However, the no-slip condition produces dead zones for mass transfer and for the stirring effect around the inner/smaller wall. On the other hand, the streamlines, which reveal that the exact fluid flow paths in the cross-section continue to increase as the *Re* increases. This results in enhanced mixing, as these secondary flows create wider/stronger vortices.

**Figure 6:**
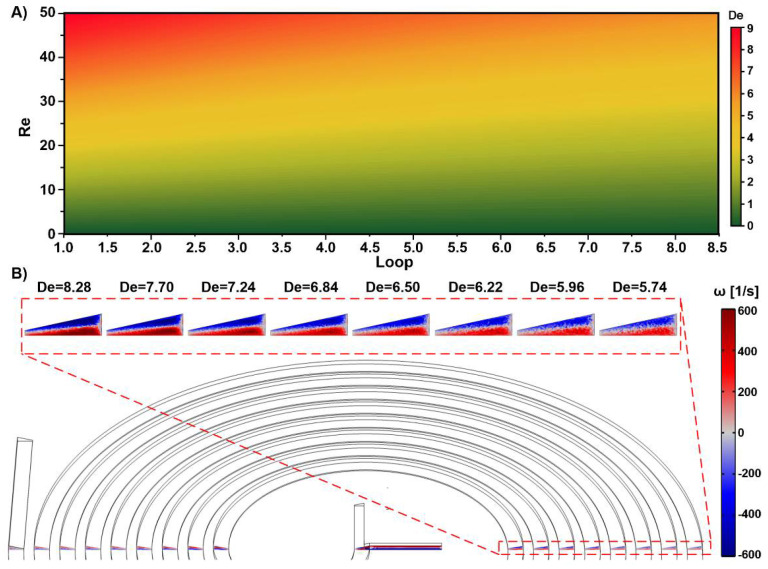
Illustration of mixing dynamics and mechanism by means of Dean flow and vortices in SM3. (**A**) The map of the Dean number is provided at Reynolds number and channel loops; (**B**) The out-of-plane component of the vorticity field at *Re* = 50 through the loops. Vortices are reduced throughout the spiral channel, and the initial loops provide better secondary flows since the dean flow grows in contrast to the curve radius.

Velocity analysis was also performed to highlight the mechanism by which the vortices and secondary flows enhance the mixing process. Figure 6A shows the Dean number map for the current design based on the Reynolds number at a loop number. It is known that the mixing efficiency improves as the *Re* rises since stronger vortices emerge, which is indicated by *De* as a manifestation of the flow characteristics. Moreover, as previously demonstrated, by increasing the velocity, the Dean number changes dramatically and is able to go through the loops; this therefore increase the changes of the fluids being properly mixed. The vortex magnitude is illustrated in Figure 6B at *Re* = 50, which shows large decreases in the strength of the vortices and in the *De* number as the fluid moves along the spiral. Thus, the primary loops offer better secondary flow effects. This is a result of the inverse relationship between the Dean flow and curvature radius. Therefore, long channels do not aid the secondary flow influence in the spiral channels, and/or fewer loops are necessary since the mixing is less dependent on the number of loops in this secondary flow state.

**Figure 7:**
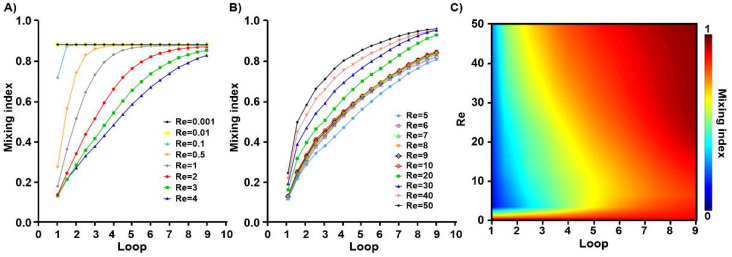
The mixing trend index for SM3 loops and Reynolds numbers. The mixing index growth over the length (**A**) before *Re* = 5; (**B**) after *Re* = 5; (**C**) the mixing contour along the spiral loops is shown for all of the applied flow rates. Very low and high velocities show proper mixing in fewer loops. No further mixing occurs after the initial loops in high *Re*, benefiting the fabrication and application requirements.

Another aspect to be considered is the required loops in each flow condition. As the flow goes through the loops of the spiral micromixer, two distinct mixing patterns were detected in the mixing index growth, one is diffusion based and one is advection based, which are depicted in Figure 7A,B. The assessment of this trend is summarized and mapped for all of the applied flow rates in Figure 7C. Contrary to 1 < *Re* < 10 flows, it can be seen that the very low and high *Re* conditions achieved proper mixing in a much smaller number of loops. In these cases, the mixing remains unchanged after only a few loops. Thus, those loops are required within these ranges, and the structure can be deliberately modified according to the application requirements that substantially reduce the input pressure usage.

## 4. Conclusions

In the present work, seven different spiral geometries were proposed and examined by experiments and using a 3D-finite element model. The nominated channel was further analyzed after finding the most efficient cross-section based on mixing performance, pressure drop, and velocity variations. The mixer with the large-angle outward trapezoidal cross-section achieved the best mixing performance and achieved the highest height aspect ratio that was studied (4.25). The mixing index of the nominated device reduced the diffusion-dominant mixing mechanism from 88% at *Re* = 0.001 to 80% at *Re* = 5. The turning point at *Re* = 5 was associated with the transition of the mixing mechanism from a diffusion to a convection mechanism. Once the convection mechanism became dominant, the mixing index reached 95% at *Re* = 50, corresponding to a pressure drop of 130 kPa. Further investigation of the loop number effect in terms of velocity and vortex emergence, which influences the mixing performance, indicated that the desired mixing rate can be achieved at a higher *Re* and by using a lower number of spiral loops. This can significantly decrease the device footprint. This study may open up new avenues for the further understanding of spiral micromixers and can be used for various applications ranging from particle synthesis to crystallization, where proper mixing and enough incubation time is necessary.

## Data Availability

The data presented in this study are available upon request from the corresponding author.
